# Calcium and vitamin D substitution for hypoparathyroidism after thyroidectomy – how is it continued after discharge from hospital?

**DOI:** 10.1007/s00423-024-03556-w

**Published:** 2024-12-05

**Authors:** Julia I. Staubitz-Vernazza, Ann-Kathrin Lederer, Nabila Bouzakri, Oana Lozan, Florian Wild, Thomas J. Musholt

**Affiliations:** Section of Endocrine Surgery, Department of General, Visceral and Transplantation Surgery, University Medical Center, Johannes Gutenberg University Mainz, Langenbeckstraße 1, D-55131 Mainz, Germany

**Keywords:** Hypoparathyroidism, Calcium supplementation, Vitamin D supplementation, Thyroidectomy

## Abstract

**Purpose:**

Postoperative hypoparathyroidism (HypoPT) is one of the most feared complications after thyroid surgery. In most cases, HypoPT is transient, requiring temporary substitution with calcium and active vitamin D. The analysis was conducted to investigate how calcium and vitamin D substitution was managed in routine postoperative clinical practice after discharge from hospital.

**Methods:**

From March 2015 to December 2023, patients with HypoPT after thyroidectomy at the university medical center (UMC) Mainz, were included in a retrospective study. The rate of continued prescription of calcium and vitamin D by external practitioners in relation to the PTH and calcium levels at the first postoperative outpatient visit at the outpatient clinic of the UMC Mainz was analyzed and critically discussed.

**Results:**

Ninety-four of 332 patients (28.3%) were continuously prescribed with calcium/vitamin D supplements: 14 had PTH deficiency and hypocalcemia and 14 had normal/elevated PTH levels with hypocalcemia, 59 had PTH values below the normal range and normo- or hypercalcemia and 7 had normal or elevated PTH levels with normocalcemia.

**Conclusions:**

There are inconsistent procedures regarding the adjustment of the calcium and vitamin D substitution by the practices providing external follow-up treatment. To avoid iatrogenic suppression of PTH levels, high calcium load and potential affection of the kidney function, a reduction scheme should be actively recommended by thyroid surgeons.

## Introduction

Hypoparathyroidism is a rare disease with a reported prevalence of about 30 patients per 100,000 [1, 2]. The vast majority of cases of chronic hypoparathyroidism is iatrogenic in origin and occurs following neck surgery [2]. However, postoperative hypoparathyroidism usually remains a temporary condition [3]. Hypoparathyroidism was defined by the European Society of Endocrinology (ESE) as “a disease with hypocalcaemia and inappropriately low parathyroid hormone (PTH) levels” [4]. Yet, in the scientific literature numerous definitions are applied, which do not adhere to the aforementioned consensus, culminating in huge differences in the reported rates of postoperative hypoparathyroidism [5]. The actual rate of postoperative hypoparathyroidism is reportedly dependent on different parameters, such as surgery-related risk factors, disease-related risk factors, patient-related risk factors and, furthermore, the perioperative calcium metabolism influences the occurrence of postoperative hypocalcemia (e.g. in case of preoperative vitamin D deficiency or hungry-bone syndrome for Graves’ disease) [6–11]. Surgery-related risk factors include extensive operations with lymphadenectomy, thyroid re-operations, difficult visualization of the parathyroid glands/parathyroid gland preservation or low surgical volume of the center [7, 9, 10]. Disease-related factors can be the presence of thyroiditis (especially Graves’ disease) or retrosternal goiter and patient-related risk factors include young age at surgery, female sex and altered activation patterns of the calcium sensing receptor [6–8, 10, 11]. To treat early postoperative hypoparathyroidism, the substitution with calcium supplements and active vitamin D analogues (alphacalcidol or calcitriol) is recommended [12]. In case of insufficient intake via an oral substitution and symptomatic hypocalcemia (carpal or pedal spasm, seizures and laryngospasm), an emergency treatment with intravenous calcium supplantation may become necessary [12]. For permanent hypoparathyroidism, active vitamin D analogues represent the most important pillar of treatment and an adequate vitamin D status with a serum concentration of 25(OH)D above 50 nmol/l should be ensured [4, 12]. The intake of calcium supplements should be reduced to a minimum: due to the lack of PTH-driven reabsorption of calcium in the distal convoluted and connecting tubule, there is an impeding risk of nephrolithiasis and nephrocalcinosis [12]. Patients with permanent hypoparathyroidism are usually referred to endocrinologists for long-term treatment and follow-up. Since most cases of postoperative hypoparathyroidism are transient, an active reduction of vitamin D and especially calcium supplements plays an important role, which usually lies in the hands of external physicians after discharge from hospital. The unquestioned continued intake of high-dosed calcium supplements may lead to an endogenous suppression of the parathyroid function, mimicking permanent hypoparathyroidism and opening the door to kidney injury due to potential calcium overload. This analysis was performed to bring the postoperative management of calcium and vitamin D substitution after discharge from hospital into the spotlight of attention.

## Materials/patients and methods

**Fig. 1:**
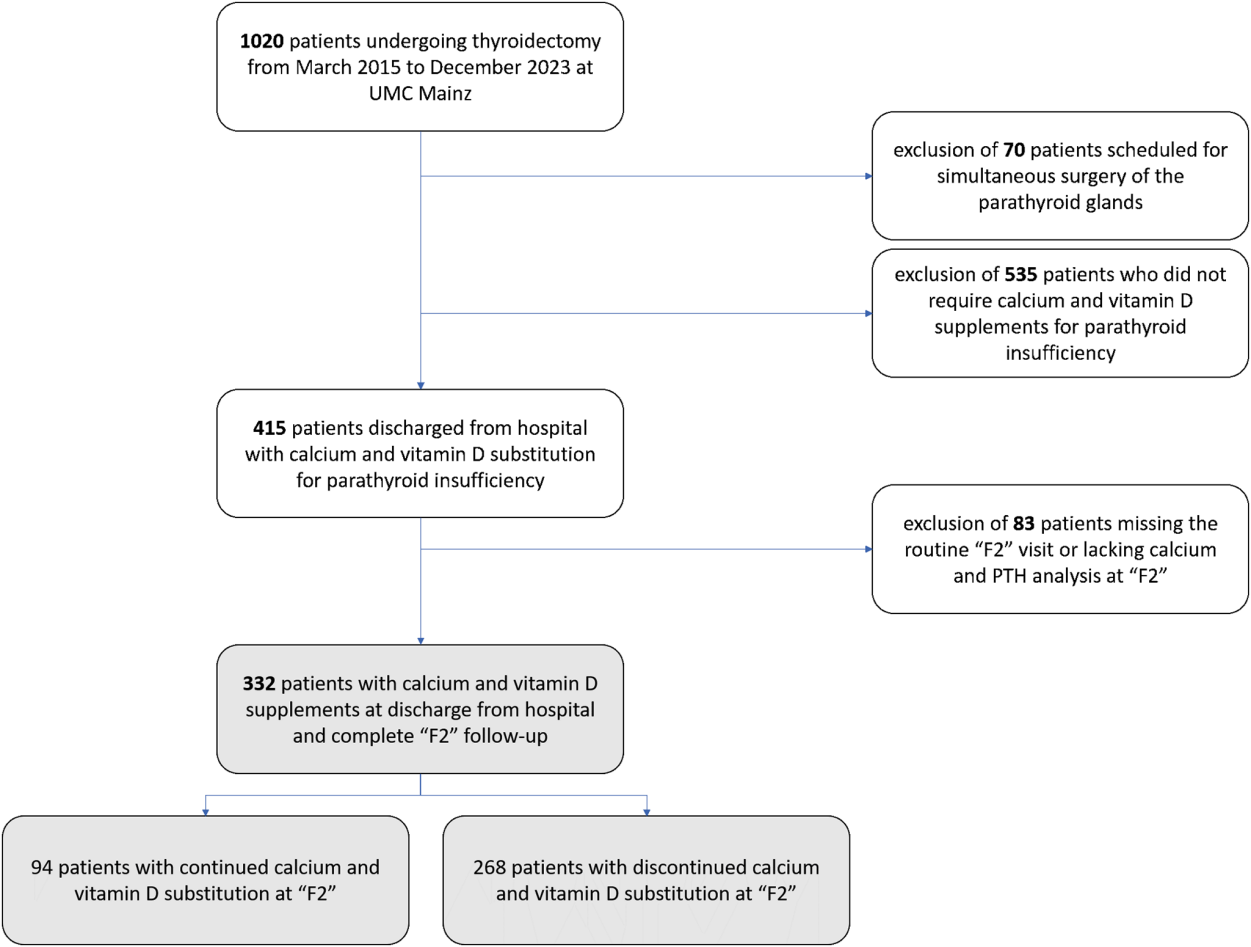
Inclusion of 332 patients treated with calcium and vitamin D supplements at discharge with complete follow-up information during the postoperative outpatient (“F2”) visit following thyroidectomy at the University Medical Center (UMC) Mainz from March 2015 to December 2023. * “2 patients with formally permanent hypoparathyroidism” = hypocalcemia and inadequately low PTH measured over 6 months after surgery, but without symptoms of hypocalcemia and no intake of supplements

In a period from March 2015 to December 2023, all patients who underwent thyroidectomy (with and without lymphadenectomy) at the University Medical Center (UMC) Mainz, Section of Endocrine Surgery who received calcium and vitamin D supplements for postoperative parathyroid insufficiency were included in this retrospective analysis. All procedures performed in studies involving human participants were in accordance with the ethical standards of the institutional and national research committee and with the 1964 Helsinki declaration and its later amendments or comparable ethical standards. The local ethical committee approved of the present study. Informed consent was obtained from all participants included in this article. Institutional patient data, extracted from EUROCRINE^®^, the European registry for endocrine surgical procedures, were reported and analyzed. Patients who simultaneously underwent scheduled surgery of the parathyroid glands (e.g. for primary or renal hyperparathyroidism) were excluded. A routine postoperative outpatient visit (“F2” visit), 3–4 weeks after discharge from hospital, was recommended to all patients undergoing thyroid surgery at the Section of Endocrine Surgery. The interval of 3–4 weeks from was deliberately set, to allow for a checkup of the correctness of the dose of L-thyroxine substitution. The “F2” visit combined a clinical examination and recommendations of further treatment with oral medication based on the laboratory parameters free triiodotyronine (fT3), free thyroxine (fT4), thyroid-stimulating hormone (TSH), calcium and PTH, which were analyzed at the day of presentation to the outpatient clinic. Patients who did not take part in the postoperative outpatient visit “F2” - or did not undergo the routine “F2” blood analysis for PTH and calcium levels - were excluded. The resulting patient cohort consisted of 332 patients (Fig. 1).

For this analysis, hypoparathyroidism was defined as hypocalcemia with inadequately low PTH, according to the guidelines on “Treatment of chronic hypoparathyroidism in adults” by the ESE [4]. The normal range for calcium values was 2.2–2.6 mmol/l in the present laboratory analyses and the normal range for PTH was 15–70 pg/ml. Hypoparathyroidism was considered permanent, if persisting for more than 6 months after surgery.

**Fig. 2:**
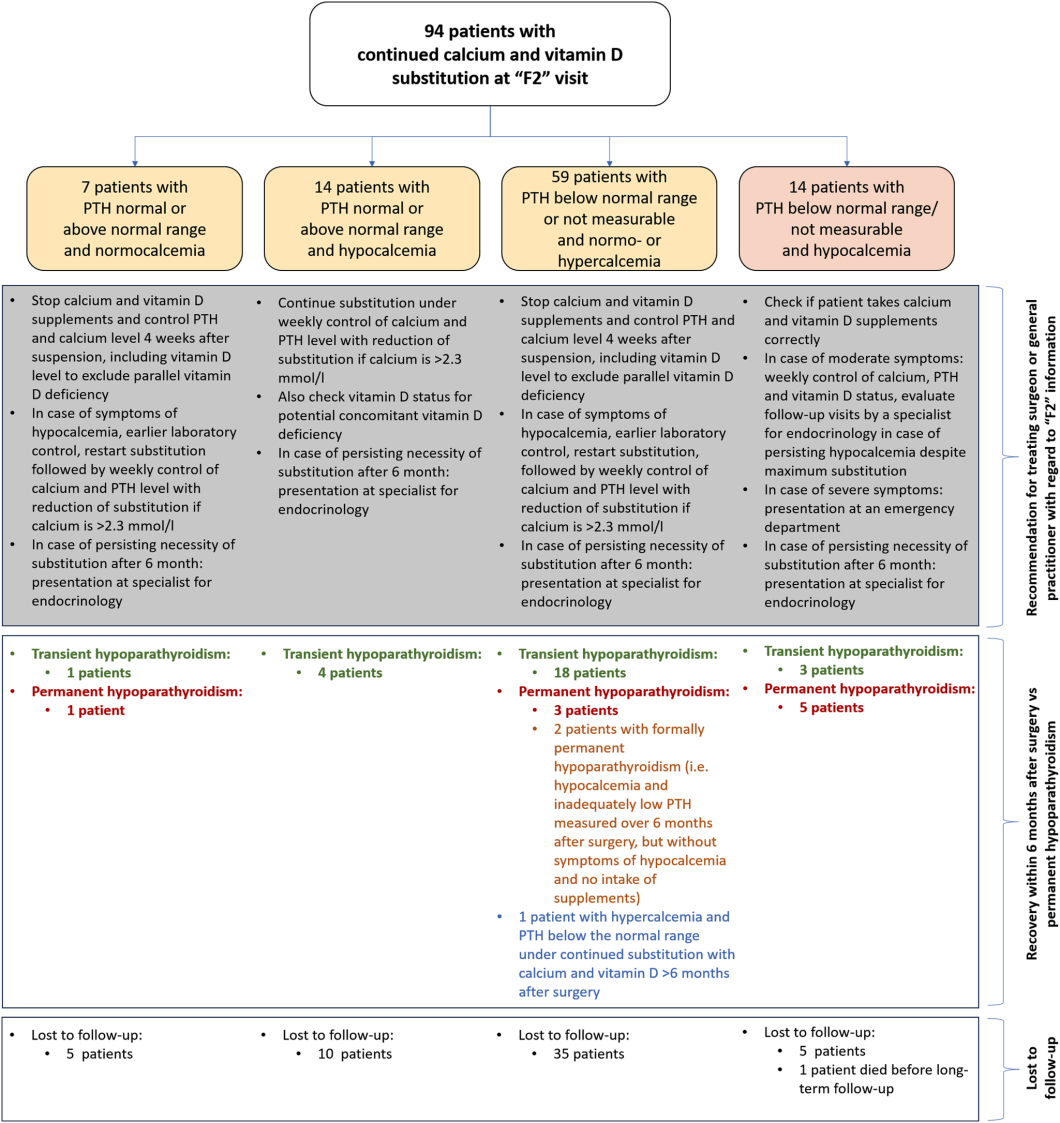
Subgroup with continued prescription of calcium and vitamin D supplements: constellation of calcium and parathyroid hormone (PTH) levels at “F2” follow up with recommended course of treatment according to the standard of the University Medical Center Mainz

The treatment scheme for early postoperative hypoparathyroidism at the Section of Endocrine Surgery at the UMC Mainz was carried out as follows: in case of asymptomatic hypoparathyroidism (hypocalcemia and PTH below normal range) and in case of mild symptoms of hypoparathyroidism (paresthesia of the extremities), an oral substitution consisting of calcium supplements (up to 1.5 g per day, divided into 3–4 doses) and calcitriol (up to 0.5 µg 3 times per day), complemented by magnesium supplements (200 mg 3 times per day) for symptomatic hypocalcemia, was administered. The aim of the substitution was a calcium level of > 1.8 mmol/l, but below 2.3 mmol/l (ensuring that the endogenous stimulus for PTH production is not impeded by a downregulation due an overdosage of calcium supplements). In cases of persistent hypocalcemia and severe symptoms such as hypocalcemic tetany, intravenous calcium infusion with calcium gluconate (10%) was administered under close laboratory monitoring of the ionized calcium in blood-gas analyses. After discharge from hospital, a weekly laboratory control of calcium and PTH levels was recommended with a monitored reduction of the calcium and vitamin D supplementation, if calcium levels above 2.3 mmol/l were measured. Discontinuation of calcium and vitamin D supplement intake was recommended, if the further monitoring revealed postoperative normo- or hypercalcemia in presence of a normalized PTH value (confer Fig. 2).

## Results

Of a total of 1020 patients undergoing thyroidectomy (malignancy rate: 31.9%, 325/1020, thyroiditis rate: 28.0%, 285/1020), 70 were excluded for simultaneously scheduled surgery of the parathyroid glands (Fig. 1). Of the remaining 950 patients, 415 (415/950, 43.7%) were discharged with a calcium and vitamin D substitution due to postoperative hypoparathyroidism. Of these, 83 abstained from the recommendation to take part in a postoperative follow-up at the outpatient clinic of the UMC Mainz (Fig. 1 and 20.0%, 83/415). 332 patients participated in the “F2” visit (Table 1, “total cohort”). However, the “F2” visit - recommended routinely 3–4 weeks after discharge from hospital - in fact took place on median postoperative day 37 (range: 3-1691 days). At the “F2” visit, 94 patients were continuously prescribed with calcium and vitamin D supplements by external practitioners (94/332, 28.3%), whereas the supplementation was suspended in 238 patients (238/332, 71.7%). The majority of patients did not undergo previous thyroid surgery (309 of the 332 patients, 93.1%), whereas 6 patients previously underwent unilateral thyroid surgery, 16 underwent bilateral thyroid surgery and one had a procedure of unclear laterality. Parathyroid reimplantation was performed in 247 of 332 patients (74.4%). Hypocalcemia with the necessity of intravenous calcium treatment during the early postoperative interval was documented in 46 of 332 patients (13.9%).

**Table 1:** Patient characteristics of the 332 patients who underwent “F2” follow-up

	Continued substitution with calcium and vitamin D (*n* = 94)	Discontinued substitution with calcium and vitamin D (*n* = 238)	Total cohort (*n* = 332)
Age (median, range)	46, 9–89	51, 3–84	49, 3–89
Sex (m: f)	32:62	75:163	107:225
Main indication for surgery			
- Malignancy (n, %) - Excluding malignancy (n, %) - Thyreotoxicosis (n, %) - Compression symptoms (n, %) - Other indications (n, %)	39,41.49 16,17.02 21,22.34 17,18.09 1,1.06	85,35.71 58,24.37 47,19.75 47,19.75 1,0.42	124,37.35 74,22.29 68,20.48 64,19.28 2,0.60
Lymphadenectomy			
- None (n, %) - Bilateral central lymphadenectomy (n, %) - Unilateral central lymphadenectomy (n, %) - Bilateral central and one-sided lateral lymphadenectomy (n, %) - Bilateral central and bilateral lateral lymphadenectomy (n, %) - Other procedure (n, %)	43,45.74 20,21.28 1,1.06 14,14.89 12,12.77 4,4.26	125,52.52 59,24.79 3,1.26 36,15.13 9,3.78 6,2.52	168,50.60 79,23.80 4,1.20 50,15.06 21,6.33 10,3.01
Histological main diagnosis			
- Malignant (n, %) o Papillary thyroid carcinoma (n, %) o Follicular thyroid carcinoma (n, %) o Poorly differentiated thyroid carcinoma (n, %) o Oncocytic thyroid carcinoma (n, %) o Medullary thyroid carcinoma (n, %) o Cancer unspecified (n, %) - Benign (n, %) o Nodular goiter (n, %) o Acute thyroiditis (n, %) o Graves’ disease (n, %) o Lymphocytic thyroiditis Hashimoto (n, %) o C-cell hyperplasia (n, %) o Follicular adenoma (n, %)	51,54.26 30,31.92 1,1.06 4,4.26 1,1.06 15,15.96 0,0.00 43,45.74 27,28.72 1,1.06 11,11.70 2,2.13 0,0.00 2,2.13	125,52.52 96,40.34 2,0.84 4,1.68 1,0.42 21,8.82 1,0.42 113,47.48 85,35.72 0,0.00 18,7.56 2,0.84 1,0.42 7,2.94	176,53.01 126,37.95 3,0.90 8,2.41 2,0.60 36,10.85 1,0.30 156,46.99 112,33.75 1,0.30 29,8.73 4,1.20 1,0.30 9,2.71
Histological secondary diagnosis^a^ (n) - Malignant (n, %) o Papillary thyroid carcinoma (n, %) o Poorly differentiated thyroid carcinoma (n, %) - Benign (n, %) o Nodular goiter (n, %) o Graves’ disease (n, %) o Lymphocytic thyroiditis Hashimoto (n, %) o Subacute thyroiditis de Quervain (n, %) o C-cell hyperplasia (n, %) o Oxyphilic adenoma (n, %) o Follicular adenoma (n, %) o “Other diagnosis” (n, %) o Incidental parathyroid adenoma (n, %) o Normal parathyroid tissue on specimen (n, %)	33 2,2.13 2,2.13 0,0.00 31,32.98 9,9.57 0,0.00 13,13.83 0,0.00 2,2.13 1,1.06 0,0.00 1,1.06 1,1.06 4,2.26	81 2,0.84 1,0.42 1,0.42 79,33.20 19,7.98 2,0.84 34,14.29 1,0.42 0,0.00 1,0.42 6,2.52 6,2.52 0,0.00 10,4.20	114 4,1.20 3,0.90 1,0.30 110,33.13 28,8.43 2,0.60 47,14.16 1,0.30 2,0.60 2,0.60 6,1.81 7,2.11 1,0.30 14,4.22
Long-term follow-up (> 6 months after surgery)			
- Permanent hypoparathyroidism (n, %)	11,11.70	9,3.78	20,6.02
o Formally permanent hypoparathyroidism but no symptoms and without intake of supplements (n, %)	2,2.13	9,3.78	11,3.31
- Transient hypoparathyroidism (n, %)	26,27.66	199,83.61	225,67.78
- Hypercalcemia and PTH below normal range in long-term follow-up (n, %)	1,1.06	0,0.00	1,0.30
- Patients lost to follow-up (n, %)	56,59.58	30,12.61	86,25.90
o Deaths within 6 months after surgery (n, %)	1,1.06	0,0.00	1,0.30

^a^ histological secondary diagnoses were present only in a portion of patients, as indicated for the subgroups/total cohort

In the subgroup with continued calcium and vitamin D intake, 14 patients had PTH deficiency and hypocalcemia (i.e. manifest hypoparathyroidism – median calcium value: 2.12 mmol/l, range: 1.85–2.19 mmol/l), 14 had normal/elevated PTH levels with hypocalcemia (illustrating an inadequate substitution for hypoparathyroidism and/or simultaneous vitamin D deficiency – median calcium value: 2.12 mmol/l, range: 1.75–2.18 mmol/l), and 7 had normal or elevated PTH levels with normocalcemia (indicating successfully treated or recovered hypoparathyroidism – median calcium value: 2.43 mmol/l, range: 2.23–2.52 mmol/l, Fig. 2).

59 patients had PTH values below the normal range and normo- or hypercalcemia, illustrating either hypoparathyroidism under substitution and/or potential cases with artificially suppressed PTH by an inadequately high calcium intake (median calcium value: 2.43 mmol/l, range: 2.20–3.07 mmol/l). Artificial suppression of PTH was particularly likely if a PTH level below the normal range and simultaneous hypercalcemia were detected (5 patients during “F2” visit). Four of these patients were lost to follow-up and in one patient hypoparathyroidism was only transient. Also, in patients with a PTH level below the normal range and normocalcemia with calcium values in the upper normal range (2.5–2.6 mmol/l, 16 patients during “F2” visit), an artificial suppression of the parathyroid function was likely. In 5 of these patients, hypoparathyroidism was transient, in one patient it was permanent and 10 patients were lost to follow-up. Generally, to avoid an artificial suppression of the PTH, a reduction or omission of the calcium and vitamin D supplementation was recommended at the “F2” visit, if the calcium value was > 2.3 mmol/l (Fig. 2).

Since long-term follow-up visits > 6 months after surgery were not clinical routine at the UMC Mainz, only in a restricted portion of the cohort long-term information about persistent hypoparathyroidism was available: permanent hypoparathyroidism was documented in 11 patients of the subgroup treated with calcium and vitamin D supplements at “F2”, including two patients who met the definition of hypoparathyroidism [4] in the long-term follow-up, but did not suffer from symptoms of hypocalcemia nor used calcium or vitamin D supplements (calcium values 2.16 and 2.19 mmol/l). Of these 11 patients, 9 had received parathyroid reimplantation during the surgical procedure. Transient hypoparathyroidism was detected in 26 cases and 56 cases remained without conclusive follow-up information. One patient with continued calcium and vitamin D intake died before long-term follow-up for permanent hypoparathyroidism. In one patient, the long-term follow-up, 25 months after surgery, revealed a hypercalcemia (2.9 mmol/l) with a PTH value below the normal range. In this case, either permanent hypoparathyroidism with inadequate calcium intake or an artificial suppression of the PTH value due to a continued superfluous intake of calcium supplements were plausible explanations for the result. Among the subgroup, there was one patient who underwent thyroidectomy with bilateral central and one-sided lateral lymphadenectomy for papillary thyroid carcinoma (pT1b, pN1b(11/32),L1,V0,Pn0,R0,M0) with an incidental parathyroid adenoma (0.8 cm in diameter), which was removed en-bloc with the specimen. Postoperative long-term follow-up (42 months after surgery) showed a normal PTH, normocalcemia and a normal 1,25(OH)_2_ vitamin D value.

**Fig. 3:**
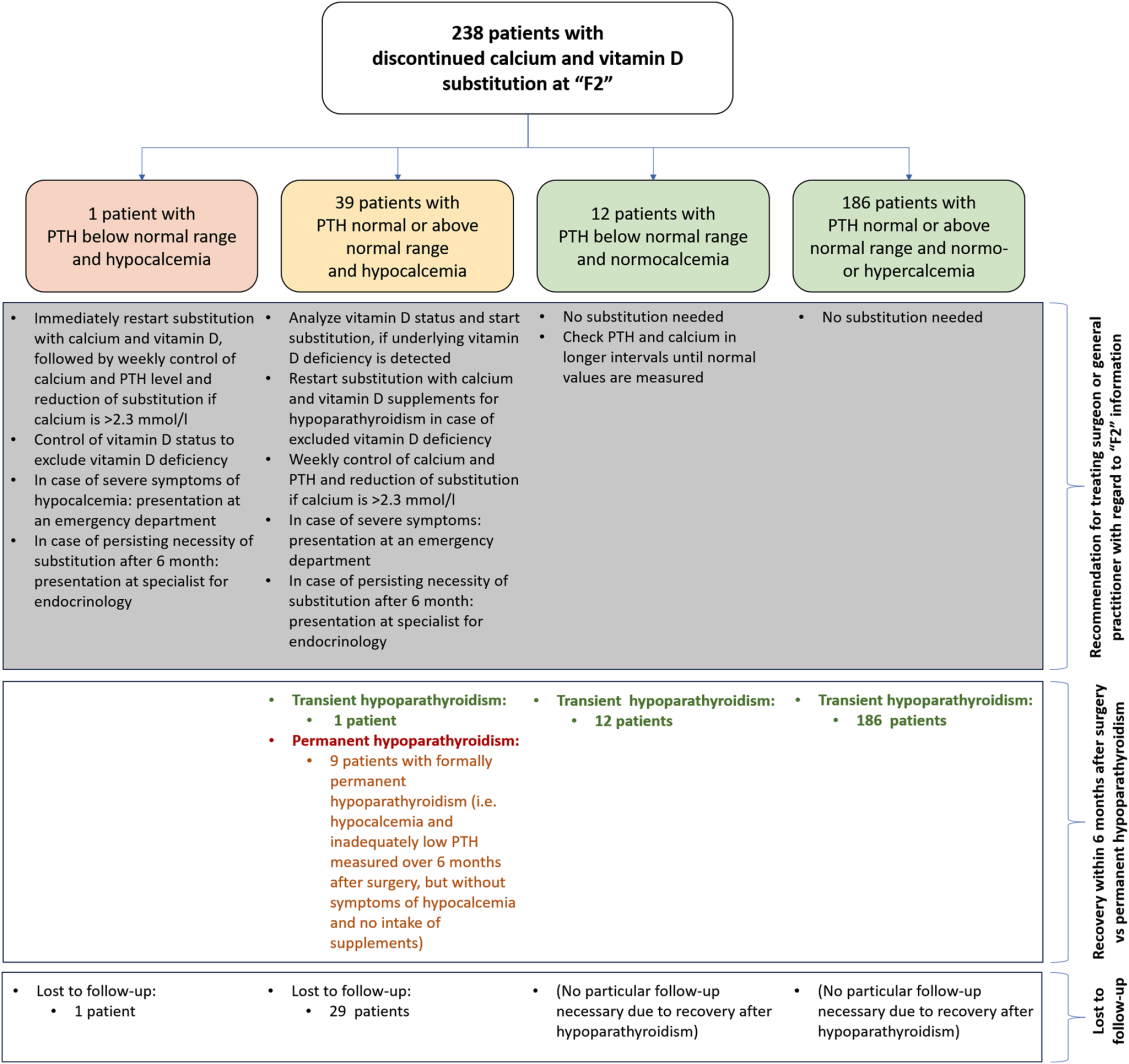
Subgroup with discontinued prescription of calcium and vitamin D substitution: constellation of calcium and parathyroid hormone (PTH) levels at “F2” follow up with recommended course of treatment according to the standard of the University Medical Center Mainz

The subgroup with discontinued calcium and vitamin D intake consisted of 238 patients (238/332, 71.7%): one had PTH deficiency and hypocalcemia (untreated hypoparathyroidism – calcium value: 2.18 mmol/l), 186 patients had normal PTH values/PTH values above the normal range and normo- or hypercalcemia (recovered hypoparathyroidism – median calcium value: 2.37 mmol/l, range: 2.21–2.68 mmol/l), 12 had PTH levels below the normal range and normocalcemia (recovered or nearly recovered hypoparathyroidism – median calcium value: 2.5 mmol/l, range: 2.24–2.58 mmol/l), 39 had normal or elevated PTH levels and hypocalcemia (hypoparathyroidism potentially complemented by vitamin D deficiency – median calcium value: 2.16 mmol/l, range: 1.7–2.19 mmol/l). In case of the documentation of a recovered postoperative hypoparathyroidism, no long-term controls were recommended. However, an elevated PTH with normocalcemia might have indirectly indicated a concomitant vitamin D deficiency, which, by recommendation, should be excluded in these cases (Fig. 3). Vitamin D deficiency might have also been present in the subgroup with normal/elevated PTH levels and hypocalcemia, which is why an analysis of the vitamin D status was recommended in these patients, too. There were 9 cases of permanent hypoparathyroidism (calcium values median: 1.15, range 1.08–2.18 mmol/l) according to the definition by Bollerslev et al. [4], however, all of these patients did not suffer from symptoms of hypocalcemia nor required calcium or vitamin D supplementation. In all of these patients, parathyroid reimplantation was performed during the surgical procedure. 30 patients of interest were lost to follow-up.

## Discussion

Postoperative hypoparathyroidism is one of the most serious complications of thyroid surgery and occurs with an increased probability following oncological operations [7–10]. Due to the postoperative outpatient treatment carried out by external practitioners, the surgical team cannot directly monitor or influence the further management of postoperative calcium and vitamin D supplementation that might be required at discharge. To address this (and other) issues, at the Section of Endocrine Surgery (UMC Mainz) a postoperative follow-up presentation is recommended as a routine check-up 3–4 weeks after surgery, including clinical visitation and laboratory analysis of the parathyroid function. The current analysis of a continued intake of calcium and vitamin D supplements at the first postoperative outpatient visit after discharge - in relation to the laboratory values of calcium and PTH - illustrated that in numerous cases either an unnecessary prescription of calcium and vitamin D supplements was to be documented, or that in case of the necessity of a continued supplementation the prescribed dosage was inadequately high. Cessation of supplementation, when it was undertaken, was rarely too early.

An exclusion of patients who underwent not only thyroidectomy but were simultaneously scheduled for surgery of the parathyroid glands was helpful to create a more homogeneous cohort for the present analysis. This was facilitated by excluding cases with an expectedly prolonged recovery interval of the parathyroid function, e.g. in patients who underwent total parathyroidectomy with partial reimplantation in case of renal hyperparathyroidism or in patients with primary hyperparathyroidism and large parathyroid adenomas with consecutive suppression of the remaining parathyroid glands. However, the relatively high portions of patients who underwent thyroid surgery for malignant main diagnoses (53.0%) and thyroidectomy with simultaneous lymphadenectomy (50.6%) act as an influential factor concerning the rate of (immediate) postoperative hypoparathyroidism of 43.7%. Parathyroid reimplantation (into the sternocleidomastoid muscle) was performed at the Section of Endocrine Surgery, UMC Mainz, in case of parathyroid glands without sufficient blood supply after thyroid surgery or if parathyroid glands were resected with the thyroid or lymphadenectomy specimen (74.4%). The recovery of the function of reimplanted parathyroid tissue is not immediate, due to the necessity of a re-establishment of sufficient nutrition after autotransplantation, but the rate of permanent hypoparathyroidism may be reduced by this technique [13–15].

Hypoparathyroidism is a complex disease which does not only refer to a deficiency of parathyroid hormone; consecutively also the level of the active form of vitamin D is reduced, since the presence of a sufficient amount of PTH is required for its synthesis [1, 2]. In addition to (symptomatic) hypocalcemia, resulting biochemical dysregulation finds expression in hypercalciuria and hyperphosphatemia, as well as an impaired quality of life with an increased risk of co-morbidities, including renal impairment and neuropsychiatric diseases [2, 3]. Due to the lack of PTH-driven reabsorption of calcium in the distal convoluted and connecting tubule of the kidneys, often in combination with the intake of calcium supplements, the risk of kidney stones in patients with chronic hypoparathyroidism is four- to eight-fold increased in comparison to the normal population [2, 16, 17]. Still, the relevance of hypoparathyroidism may, due to the indirect character of influence, be underestimated. An active surveillance of the recovery of postoperative hypoparathyroidism is necessary to understand, in how far a calcium and vitamin D supplementation is needed, and in which cases a superfluous intake might interfere with the endogenous restoration of the calcium homeostasis and even lead to serious complications. In this sense, another important aspect that has to be considered - especially for the treatment with calcium supplements - is that the calcium-sensing receptor (CaSR), a plasma membrane–bound receptor, is activated by extracellular calcium [18, 19]. The CaSR is expressed in multiple tissues including parathyroid gland, thyroid, kidney, intestine, bone, bone marrow, brain, skin, pancreas, lung, and heart [18, 20–25]. In a physiological setting, activation of parathyroid gland CaSR regulates the calcium homeostasis by suppressing PTH secretion [18]. The unquestioned intake of high-dosed calcium supplements, therefore, may consequently lead to an endogenous suppression of the parathyroid function, mimicking permanent hypoparathyroidism (by the result of a low PTH level in blood samples) and opening the door to kidney injury due to a potential calcium overload at the same time. In this study, at the “F2” visit, there were 5 patients with PTH below the normal range and hypercalcemia and 16 with PTH below the normal range and calcium in the upper normal range (2.5–2.6 mmol/l). In these patients, following “F2” presentation, it was recommended to particularly monitor the calcium level and actively reduce the calcium and vitamin D intake according to the aim of a calcium level < 2.3 mmol/l and, in addition, further controls in the outpatient clinic of the UMC Mainz were optionally recommended. Similarly, analyses by other German working groups revealed that calcium weaning in case of recovered postoperative hypoparathyroidism in the outpatient setting was insufficient [26, 27]. Generally, for hypercalcemia with normal PTH values, various differential diagnoses must be taken into consideration, including osteolysis for bone metastases of cancer, granulomatous diseases, medication with influence on calcium excretion or familial hypocalciuric hypercalcemia (FHH, a rare genetic disease which leads to a reduced sensitivity of the parathyroid CaSR to elevated calcium levels) [28–30]. In turn, a solitary elevation of PTH without concomitant hypercalcemia may not only result from secondary hypoparathyroidism (caused by vitamin D deficiency and/or chronic renal failure), but also from primary hyperparathyroidism at an initial/compensated state [28, 31]. Furthermore, critical anamnesis should include the questions for an excessively high intake of sodium or magnesium as well as for medication including thiazide diuretics, lithium carbonate and antiacids, which might result in altered calcium values [28].

Even though hypoparathyroidism is generally considered permanent if persisting for more than 6 months after surgery, there are numerous patients who experience a late recovery after significantly longer intervals, which must be taken into consideration during follow-up visitations [32, 33]. For permanent hypoparathyroidism, it was recommended by the ESE to keep the serum calcium level in the lower normal range and active vitamin D analogues represent the first-line treatment [4, 12]. The intake of calcium supplements was recommended to be reduced to a minimum, and should be administered only if dietary intake of calcium-rich nutrition does not allow for a sufficient calcium level without the symptoms of hypocalcemia [12]. Of the patients with a long-term control in the present cohort, in whom postoperative hypoparathyroidism was considered permanent, there were 2 patients with a combined intake of calcium and vitamin D supplements, in one case with severe hypercalcemia, which is against the recommendation of the ESE to keep the calcium level in the lower normal range for nephroprotection [4]. In the present cohort, there were 11 patients who met the criteria for the definition of permanent hypoparathyroidism (hypocalcemia with inadequately low PTH over 6 months after surgery [4]), but did not suffer from symptoms of hypocalcemia and were not prescribed with calcium or vitamin D supplements. Generally, no data exist concerning the exact optimal calcium concentration during the treatment of hypoparathyroidism [12]. Therefore, calcium values slightly under the normal range may be tolerated. In rare cases, genetic mutations or an overactivation of the CaSR by autoantibodies, an excessive intake of magnesium or other medications may indirectly reduce PTH and consecutively calcium values in certain patients [11, 34].

However, for these patients, a presentation at an endocrinologist should be scheduled, for a verification of the presumed diagnosis of permanent hypoparathyroidism and a lifelong monitoring/treatment in case of its confirmation.

As a drawback of the present study, due to different reasons, the recommended interval of 3–4 weeks between the operation and the “F2” visit was often stretched to a longer period between surgery and the actual follow-up visit (“F2” on median postoperative day 37, range: 3-1691 days). Worse still; 20.0% of patients (83/415) discharged with calcium and vitamin D supplements for hypoparathyroidism after thyroidectomy totally abstained from the recommendation to take part in a postoperative follow-up at the outpatient clinic of the UMC Mainz. The patients themselves and/or the general practitioners and specialists in nuclear medicine in charge of the follow-up care were contacted by our hospital, but, yet, without success in the mentioned cases. Patients’ personal commitments, long journeys, an acute shortage of available appointments at attractive times and, above all, the lack of subjective complaints in connection with possible postoperative complications were reasons for patients to forego (repetitive) postoperative visits to our outpatient clinic. Since this retrospective study depicts the reality of care in a German high-volume center, it shows that the cost structure and the structure of the German healthcare system itself make follow-up care considerably more difficult. A prospective controlled study - on the other hand - would most probably not have revealed this deficit, as there is a greater obligation for patients to return for follow-up care within such a study.

Another general drawback of the present analysis is that the calcium value at the “F2” visit is only analyzed once in a specific time point, and the result is dependent on the time of calcium intake and biological fluctuations. Hypocalcemia may be masked by calcium intake shortly before the measurement, whereas a longer pause between calcium intake and blood analysis results in the picture of a more severe hypocalcemia. Moreover, the vitamin D status is not routinely analyzed during the follow-up visit. Therefore, information on potential concomitant vitamin D deficiency were not routinely available at the “F2” visit, but it was recommended that an analysis of active vitamin D concentration should be performed with outside physicians if calcium and PTH levels indirectly indicate possible vitamin D deficiency. In patients with permanent hypoparathyroidism, in addition to an analysis of the free-ionized or albumin-adjusted calcium concentrations every 3–6 months, the ESE recommended monitoring of phosphate/the calcium-phosphate product, magnesium and the renal function (including 24-h urinary calcium and creatinine excretion in intervals from 6 to 12 months) [12]. Since the treatment of permanent hypoparathyroidism should be guided by a specialist in endocrinology, also the complex monitoring should lie in the hands of endocrinologists, even though this may be complicated in the reality of care in rural areas.

To facilitate the adequate reduction of calcium and vitamin D supplementation for presumed transient postoperative hypoparathyroidism, from 2018 onward, in the demission report of the Section of Endocrine Surgery, UMC Mainz, an active reduction of the calcium and vitamin D supplementation was openly recommended in accordance with the practice guidelines of the German Association of Endocrine Surgeons for the surgical treatment of benign thyroid disease [35, 36]. It was further recommended, to perform a reduction of the supplements in case of calcium levels > 2.3 mmol/l in a weekly control, to avoid a calcium overload and iatrogenic suppression of the parathyroid function.

## Conclusions

Concerning postsurgical hypoparathyroidism, the role of the operating surgeon is, in addition to an optimization of the parameters which are useful to avoid hypoparathyroidism in the first place (i.e. during surgery), to indicate to the treating general physicians how the medication for (transient) postoperative hypoparathyroidism should be reduced. Moving forward, patients with permanent hypoparathyroidism must be differentiated from those with artificial suppression of parathyroid function by an unnecessary intake of calcium and vitamin D supplements. These patients should be referred to endocrinologists for further verification of the diagnosis, followed by lifelong monitoring and treatment of this rare but complex condition, if permanent hypoparathyroidism is confirmed.

## Data Availability

No additional datasets were generated or analysed during the current study.
